# Bioinformatical dissection of fission yeast DNA replication origins

**DOI:** 10.1098/rsob.200052

**Published:** 2020-07-22

**Authors:** Koji Masuda, Claire Renard-Guillet, Katsuhiko Shirahige, Takashi Sutani

**Affiliations:** Institute for Quantitative Biosciences, The University of Tokyo, 1-1-1 Yayoi, Bunkyo-ku, Tokyo 113-0032, Japan

**Keywords:** replication origins, pre-replication complex, fission yeast, ChIP-seq, machine learning

## Abstract

Replication origins in eukaryotes form a base for assembly of the pre-replication complex (pre-RC), thereby serving as an initiation site of DNA replication. Characteristics of replication origin vary among species. In fission yeast *Schizosaccharomyces pombe*, DNA of high AT content is a distinct feature of replication origins; however, it remains to be understood what the general molecular architecture of fission yeast origin is. Here, we performed ChIP-seq mapping of Orc4 and Mcm2, two representative components of the pre-RC, and described the characteristics of their binding sites. The analysis revealed that fission yeast efficient origins are associated with two similar but independent features: a ≥15 bp-long motif with stretches of As and an AT-rich region of a few hundred bp. The A-rich motif was correlated with chromosomal binding of Orc, a DNA-binding component in the pre-RC, whereas the AT-rich region was associated with efficient binding of the DNA replicative helicase Mcm. These two features, in combination with the third feature, a transcription-poor region of approximately 1 kb, enabled to distinguish efficient replication origins from the rest of chromosome arms with high accuracy. This study, hence, provides a model that describes how multiple functional elements specify DNA replication origins in fission yeast genome.

## 1. Background

DNA replication in eukaryotes initiates bidirectionally at multiple distinct sites in the genome called DNA replication origins. A great deal of work over the last few decades has revealed how the pre-replication complex (pre-RC) is assembled and activated at the origins to ensure concordant initiation of DNA replication during the cell cycle [1–4]. First, origin recognition complex (Orc) recognizes and binds the origin sites. The bound Orc then recruits the DNA replicative helicase Mcm (consisting of the Mcm2-7 hexameric complex) onto DNA with the help of the Cdc6 and Cdt1 proteins, which are functional only in G1 phase. At the onset of S phase, phosphorylation by Cdk and Ddk kinases triggers recruitment of several additional factors, including DNA polymerases, onto the pre-RC, leading to formation of an active replicative complex and the initiation of replication. Despite a deep understanding of *trans*-acting factors required for ‘once and only once’ replication initiation during the cell cycle, the nature of the *cis*-acting elements that define the origins remains to be elucidated in most eukaryotes.

The most well-studied replication origins are those in the budding yeast *Saccharomyces cerevisiae*. They are relatively short (approx. 200 bp) and contain an 17 bp T-rich DNA motif called the ARS consensus sequence (ACS) [3,5] that is recognized by Orc *in vitro* and *in vivo* and is essential for origin activity [6,7]. However, because potential ACS matches in the genome are far more abundant than functional origins, the ACS is not sufficient to specify origin location in the budding yeast genome [8,9]. Another feature associated with budding yeast replication origins is a nucleosome-free region (NFR) [10,11]. It is typically approximately 125 bp long, and the ACS motif is located asymmetrically within it. NFR formation is likely to be dependent on a stretch of A residues located downstream from the ACS, which resembles B elements reported in several origins. It is widely believed that the ACS asymmetrically located within an NFR specifies location of replication origin in budding yeast.

By contrast, determinants of replication origins in other eukaryotes, particularly in metazoans, remains enigmatic. Recent genome-wide studies failed to identify any clear consensus DNA motifs associated with origins in *Drosophila* and human [12,13]. Instead, GC-rich sequences that can form G-quadruplexes were frequently identified in the vicinity of the origins ranging from *Drosophila* to humans [5,14], although this signature, on its own, is not sufficient to define an origin location within the genome. Bioinformatics analyses also revealed that DNA accessibility and several histone modifications representing active chromatin are associated with origins, and these features in combination can predict origin position with moderate accuracy [12,13,15]. However, it has not been elucidated yet whether these epigenetic marks are indeed required features of origins, and if so, what other features are necessary to fully determine origin location.

Replication origins of the fission yeast *Schizosaccharomyces pombe* are thought to resemble those of metazoans more closely than those of *S. cerevisiae*, in that they are relatively long (greater than 500 bp) and contain no single essential consensus sequence like ACS [16]. Functional dissection of strong replication origins revealed that each of them contains one or two DNA regions essential for origin function [16–18]. These regions are all asymmetric AT-rich sequences of 30 bp or longer. Such poly(dA) tracts are shown to be a binding sequence of fission yeast Orc4 protein *in vitro* and *in vivo* [19–23]. However, sequences similar to the poly(dA) tracts are not necessarily found in other origins [24–26], and it remains unclear how divergent the binding sequence of Orc4 is among origins in the genome. Genome-wide analysis demonstrated that AT-richness is a good predictor of replication origins in fission yeast [25,26]; 500–1000 bp-long DNA regions with distinctively high AT content tend to be colocalized with origins in 90% of the cases tested [25]. However, it remains to be understood how this attribute leads to pre-RC formation, and whether any feature other than the poly(dA) tract plays a role in origin specification.

In this study, we conducted genome-wide high-resolution mapping of pre-RC components in fission yeast, and performed in-depth bioinformatics analysis of the identified genome locations. The analysis revealed three DNA-encoded features associated with the pre-RC binding sites and suggested independent contribution of each feature to pre-RC assembly. The identified features, in combination, allowed accurate computational prediction of pre-RC sites in the genome, implying that these three features are the major *cis*-determinants of DNA replication origin in fission yeast.

## 2. Methods

### 2.1. Yeast strains

All yeast strains used in this study are listed in electronic supplementary material, table S1. Yeast strains were grown in complete YPD [27]. Tagging of endogenous *orc4*^+^ and *mcm2*^+^ genes with nine copies of the PK epitope was carried out by plasmid integration, as previously described [28]. To invert the direction of the *def1^+^* and *urg1*^+^ genes, the endogenous gene was replaced with a DNA fragment containing the corresponding gene in an inverted direction as described in [29]. Epitope tagging and gene inversion were verified by immunoblotting and colony PCR, respectively.

### 2.2. Cell synchronization

To arrest cells in G1 phase, *cdc10-V50* cells [30] were cultured in YPD at 26°C to a density of 5–6 × 10^6^ cells ml^−1^, shifted to a restrictive temperature of 36°C and incubated for 3.5 h. To identify early-replicating genome regions by DNA copy number analysis, cells arrested in early S and G2 phases were prepared. *cdc25-22* cells [31] cultured in YPD at 26°C to a density of 5–6 × 10^6^ cells ml^−1^ were shifted to a restrictive temperature of 36°C, and then incubated for 3.5 h to arrest them in late G2 phase. Half of the cells were then released into YPD containing 11 mM hydroxyurea, and incubated at 26°C for 2.5 h to arrest them in early S phase [27]. Synchronization was assessed by flow cytometry analysis of DNA content [27].

### 2.3. Chromatin immunoprecipitation sequencing

Chromatin immunoprecipitation (ChIP) was carried out as described in [28], by using the PK epitope tag [32] for immunoprecipitation. Subsequently, DNA before and after ChIP was processed and sequenced on an Illumina HiSeq 2500 instrument, yielding 51 bp-long single-end reads. The obtained reads were then mapped against *S. pombe* reference genome sequence (ASM294v2.19) [33] using Bowtie2 (version 2.1.0) with default parameters [34]. Information and statistics regarding sequencing and mapping are summarized in electronic supplementary material, table S2. The mapping results were fed into the DROMPA2 chromatin immunoprecipitation-sequencing (ChIP-seq) analysis package (version 2.5.1) [35] to generate a ChIP-seq profile or a list of fold-enrichment (FE) ratios for each 10 bp genomic bin. Peak calling was also performed in DROMPA2 with default parameters, except that -ipm was set to 6 (for Orc4) or 2 (for Mcm2). In this study, only ChIP-seq peaks located on chromosome arms were analysed; the peaks in centromeric, telomeric and rDNA gene regions were omitted from the subsequent analysis. Peaks whose widths were smaller than 50 bp were judged to be false-positive signals and discarded. When multiple peak summits were called in a single, consecutive region with FE higher than the threshold, the entire region was considered as a single peak, and the summit was assigned to the highest component peak. The number of resultant highly confident ChIP-seq peaks on chromosome arms was 714 for Orc4 and 337 for Mcm2. The median peak widths for Orc4 and Mcm2 are 534 bp and 569 bp, respectively. When an Orc4 peak had a 1 bp or larger overlap with a Mcm2 peak, the corresponding Orc4 peak was considered as a region where Orc4 and Mcm2 proteins co-localized, and referred to as an OM site. Other Orc4 sites that had no overlap with any Mcm2 peaks were referred to as O sites. To map Mcm4 binding sites in *hsk1* cells, ChIP-seq data in SRA (SRR1773448) were used [36].

### 2.4. Chromatin immunoprecipitation-quantitative PCR

Quantitative PCR of ChIP-purified DNA (ChIP-qPCR) was carried out as described in [28], using primer sets listed in electronic supplementary material, table S3.

### 2.5. DNA copy number analysis

HiSeq sequencing data of Mcm2 ChIP-seq input sample was used to verify that no DNA replication initiated in *cdc10-V50* cells used for ChIP-seq. As controls, genomic DNA was isolated from fixed *cdc25-22* cells arrested in early S and G2 phases, and sequenced and analysed as described in the ‘ChIP-seq’ section. DNA copy number of G1 and early S phase cells, relative to G2 cells, was calculated for each 100 bp genomic bin and smoothened by LOESS smoothing with a span of 0.005 [24]. Finally, the whole set of ratios was scaled so that the 25% percentile was equal to 1.

### 2.6. Motif analysis

MEME (version 4.11.1) [37] and DME2 [38] were used to perform motif discovery around the OM and O sites. Five hundred base pair DNA fragments centred on each Orc4 ChIP-seq peak summit were used as query sequences. The used parameters were ‘-mod anr -minw 6 -maxw 15 -revcomp’ for MEME, and ‘-n 100 -w 15’ for DME2. In both cases, non-coding DNA sequences (that is, intergenic and intronic sequences) were used as a background to compensate for AT content. The FIMO algorithm (in MEME Suite 4.11.4) [37] was used to search DNA sequences in the genome that matched the motifs detected by MEME and DME2, using 5% FDR as a threshold. The resultant lists of MEME- and DME2-based motif locations were combined to generate a list of poly(dA) motif locations in the genome. If multiple locations in the list overlapped, they were united into a single, larger site.

### 2.7. Calculation of free energy for DNA strand separation

Free energy required for DNA strand separation (ΔG_melt_) was calculated in a sliding window of 200 bp using the nearest-neighbour model described in [39].

### 2.8. Gene annotations

Gene annotations were from release v57 of PomBase. In this work, ‘genes’ indicates protein-coding genes, excluding dubious open reading frames (ORFs). For genes with no untranslated region (UTR) information, gene start and end sites (transcription start and termination sites, respectively) were estimated based on the corresponding ORF coordinates and genome-wide mean length of 5′ UTR and 3′ UTR (293 bp and 430 bp, respectively). IGRs (intergenic regions) were defined as the regions on chromosome arms and not contained in any of the genes.

### 2.9. RNA-seq

RNA-seq data from cells in G1 phase (GSM1262382) [40] were analysed to estimate transcriptional activity at each locus. Sequenced reads were aligned to *S. pombe* reference sequence using TopHat2 version 2.0.9 [41]. The total number of mapped reads was approximately 5.7 million. The expression level for each gene was calculated as reads per kilobase of exon per million mapped reads (RPKM) using an in-house script.

### 2.10. Machine learning-based analysis

Support vector machines (SVMs) with the RBF kernel [42] were used to discriminate IGRs coinciding with OM sites (*n* = 276) from OM site-negative IGRs (*n* = 3036). The kernlab package [43] in the R statistical computing environment [44] was used to construct the SVMs. A set of features (described in electronic supplementary material, figure S2a) were scored for a 1000 bp window centred on each of the Orc4 ChIP-seq peak summits located in IGRs (for OM site-positive IGRs) or the midpoint of each IGR (for OM site-negative IGRs), based on DNA sequence and RNA-seq data of the region. These features were then used, alone or in combination, to build SVMs. The hyper-parameters *σ* and C were tuned by a grid search and set to 0.01 and 10, respectively. Probabilistic outputs for SVMs were calculated as described in [45]. Performances of SVM classifiers were estimated by computing the area under the curve (AUC) of the receiver operating characteristics (ROC) curve and precision-recall (PR) curve, using ROCR and PRROC packages in R, respectively [46,47]. Evaluation of classifiers was conducted by fourfold cross validation (CV): the dataset was divided with stratification into four subsets, one of which was used in each trial as a test set to evaluate the classifier trained using the rest of the dataset. The mean AUC (for ROC or PR) was calculated from the four AUC values obtained in a single round of fourfold CV. This analysis was repeated 10 times using different partitions of the dataset, and then the mean and 95% confidence interval of the mean AUCs were calculated. To apply the classification models to intragenic OM sites, we generated a dataset consisting of the intragenic OM sites (*n* = 27) and control genomic sites that were randomly chosen from the genome and greater than 1 kb away from any OM or O sites (*n* = 135). Three genetic features, *N*_mt_, *L*_AT_ and *L*_ntrx_ (electronic supplementary material, figure S2a), were scored for 1000 bp windows centred on Orc4 ChIP-seq peak summits (in intragenic OM sites) or the control sites. These features were fed into each classifier built in the iterated fourfold CV described above, to evaluate classifier performance. F-score used for feature selection is described in [48].

### 2.11. Statistical analysis

Two-sided Mann–Whitney *U*-test was used for statistical analysis. Confidence intervals were calculated by the percentile bootstrap.

## 3. Results

### 3.1. High-resolution mapping of fission yeast pre-replication complex sites

To reveal DNA sequence signatures associated with Orc and Mcm-binding sites in fission yeast replication origins, we determined genome-wide binding locations of pre-RC components, Orc4 and Mcm2, at high resolution by epitope tag-based ChIP-seq. We used *cdc10-V50* temperature-sensitive mutation to arrest cells in G1 phase. Cdc10 encodes a subunit of MBF transcription factor, which is required for induction of Cdc18 (*S. pombe* Cdc6 orthologue) and Cdt1 [49,50], and it is widely believed that no pre-RC is formed in *cdc10*-arrrested cells. We, however, found that pre-RC was actually formed at a significant number of genome locations in *cdc10*-arrested cells, and these sites consisted largely of highly efficient origins as shown below.

**Figure 1.**
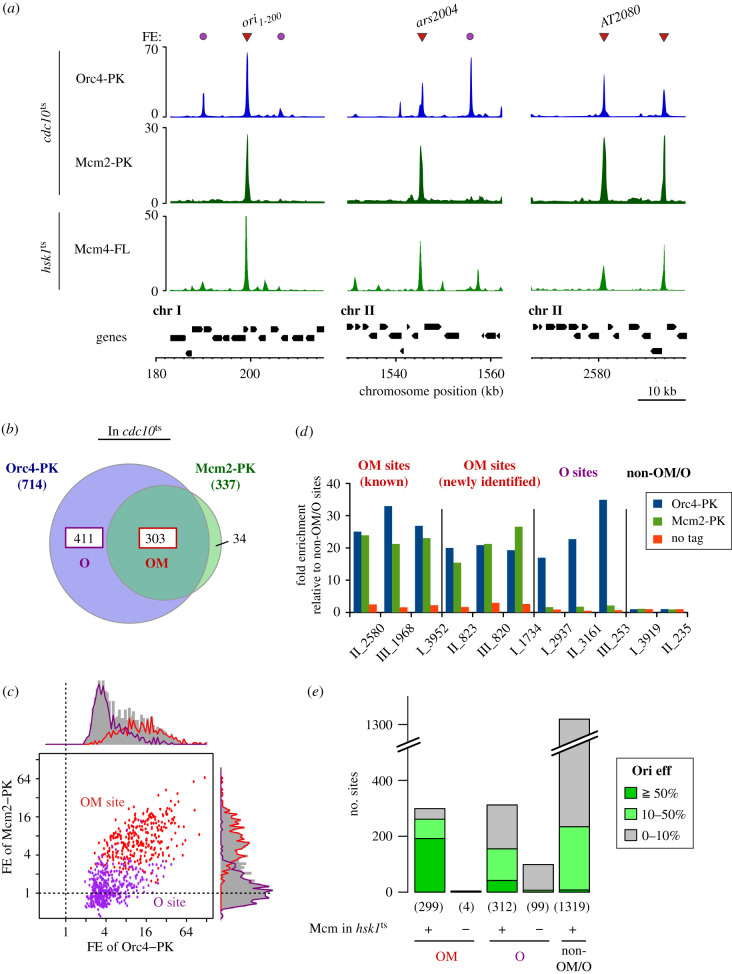
Identification of pre-RC- and Orc-only-binding sites in the fission yeast genome. (*a*) ChIP-seq profiles of PK-tagged Orc4 (Orc4-PK) and Mcm2 (Mcm2-PK) in *cdc10*-arrested cells. The *y*-axes show FE. Three representative genome regions, including well-studied replication origins (ori1–200, ars2004 and AT2080), are shown. Red arrowheads indicate sites where both Orc4 and Mcm2 were co-localized (OM sites), and magenta circles sites where only Orc4 was localized (O sites). The third row indicates Mcm4-FLAG ChIP-seq profile in *hsk1* mutant [36]. The bottom row (Genes) shows position, size and direction of transcriptional units. (*b*) Venn diagram indicating overlap between Orc4-PK- and Mcm2-PK-binding sites detected on chromosome arms. (*c*) Dot plot representation of Orc4-PK and Mcm2-PK FEs at each OM (red) or O (purple) site. Black dotted lines indicate a FE of 1, i.e. no enrichment in ChIP isolated DNA. Distributions of Orc4-PK and Mcm2-PK FEs are shown on the upper and right sides of the dot plot, respectively. (*d*) Validation of Orc4-PK- and Mcm2-PK-binding sites by quantitative PCR measurement of DNA co-immunoprecipitated with Orc4-PK (blue) and Mcm2-PK (green). ‘no tag’ (orange) indicates a control experiment in which cells without any epitope tag were subjected to anti-PK chromatin immunoprecipitation. DNA corresponding to OM sites, O sites or sites without Orc4 or Mcm2 binding (non-OM/O sites) was quantified. The qPCR locus name represents chromosome number (Roman numerals) and coordinate (Arabic numerals following underscore in kb). FEs of ChIP-purified DNA at the indicated loci are shown relative to the average value at the non-OM/O sites. (*e*) The number of sites with ≥50% (dark green), 10–50% (light green) and less than 10% (grey) relative origin efficiency (Ori Eff) [51] in each indicated class of genomic sites. + and – indicate the presence and absence of Mcm4 peak in *hsk1* cells, respectively. A number in parentheses indicates the total number of the sites belonging to the indicated class.

*cdc10-V50* cells expressing PK epitope-tagged Orc4 or Mcm2 were arrested in G1 phase and subjected to anti-PK ChIP-seq analysis. Binding sites within centromere, telomere and rDNA sequences were omitted from our analyses because these regions are in distinct chromatin environments. After peak calling and further processing, we obtained 714 peaks for Orc4 and 337 for Mcm2 (figure 1*a,b*). The Orc4 and Mcm2 peaks overlapped at 303 sites, which are hereinafter referred to as ‘OM sites’. These sites presumably correspond to pre-RC-binding sites in *cdc10*-arrested cells. In addition, we detected on chromosome arms a significant number (411) of sites at which only Orc4 protein was localized (O site) (figure 1*a,b*). Contrarily, we observed a far fewer number (37) of sites where Mcm, but not Orc, was localized (figure 1*b*), which is consistent with the fact that binding of Orc to origins is a prerequisite for Mcm helicase loading. ChIP-seq peak height, or FE of Mcm2, showed a bimodal distribution, and one of the peaks that centred around one (that is, no enrichment) corresponded to the O sites (figure 1*c*). We also conducted quantitative PCR measurement of ChIP-purified DNA (ChIP-qPCR) at several selected locations (six and three for OM and O sites, respectively), and confirmed binding of Orc4 and Mcm2 to the OM sites, and binding of Orc4, but not Mcm2, to the O sites (figure 1*d*). Thus, the O sites are neither false-positive peaks of Orc4 nor missing Mcm2 peaks, and the OM and O sites are likely to be distinct classes of sites in the genome.

The detected Mcm2 peaks may be derived from a fraction of cells that are outside G1 phase, and not reflect a genuine pre-RC. Flow cytometry analysis, however, confirmed that most, if not all, of the cells were arrested in G1 phase (electronic supplementary material, figure S1a). DNA copy number analysis also revealed no sign of replication initiation around the Orc-binding sites in the cells used for Mcm2 ChIP-seq (electronic supplementary material, figure S1b).

Previously, Kanoh *et al*. [36] conducted Mcm4 ChIP-seq in *hsk1* mutant, in which Cdc18 and Cdt1 are fully induced, but no DNA replication is initiated. Our ChIP-seq analysis pipeline identified 1880 Mcm4 peaks in the *hsk1* mutant dataset (figure 1*a*). We compared the OM and O sites detected in *cdc10*-arrested cells with the Mcm4 binding sites in *hsk1*. Among 303 OM sites, 299 (99%) were colocalized with Mcm4 peaks in *hsk1* (figure 1*e*). The Mcm peaks in the two conditions overlapped precisely, and the median peak summit difference at the OM sites was 99 bp. We also found that, among 411 O sites, 312 (76%) were colocalized with Mcm4 peaks in *hsk1* (figure 1*e*). These results indicate that almost all OM sites reflect genuine pre-RC binding sites, and that Mcm loading would occur at the majority of the O sites after induction of Cdc18 and Cdt1 proteins.

We, then, assessed replication initiation activity at the OM and O sites, using a publicly available dataset. Daigaku *et al*. [51] identified location and measured initiation efficiency of replication origins in fission yeast genome by polymerase usage sequencing (Pu-seq) technology. Among 299 OM sites with Mcm4 binding in *hsk1*, 192 (64%) were colocalized with strong (i.e. ≥ 50% relative origin efficiency) replication origins, and 69 (23%) with weak (i.e. 10–50% efficiency) ones (figure 1*e*). By contrast, the O sites are less efficient in replication initiation. Among the O sites to which Mcm4 was loaded in *hsk1*, 13% and 36% were colocalized with strong and weak origins, respectively, and only 7% of the O sites that had no Mcm4 loading in *hsk1* were associated with strong or weak replication origins (figure 1*e*). Comparison with well-studied replication origins confirmed the above findings; among 28 origins previously shown to be active *in vivo* by two-dimensional gel analysis (listed in [52]), 25 (89%) were co-localized with the OM sites. We also noticed that among the Mcm4 binding sites in *hsk1* cells that are overlapped with neither OM nor O sites, 82% showed little or no initiation activity (figure 1*e*). Pre-RC assembly seems inefficient or incomplete at these sites. Taken together, we conclude that the OM sites consist largely of the genome regions that are capable of supporting efficient pre-RC assembly. ChIP-seq data provides high-resolution binding profiles of Orc4 and Mcm2 within the OM sites, which were used in the following analysis.

### 3.2. DNA motifs associated with Orc binding sites

**Figure 2.**
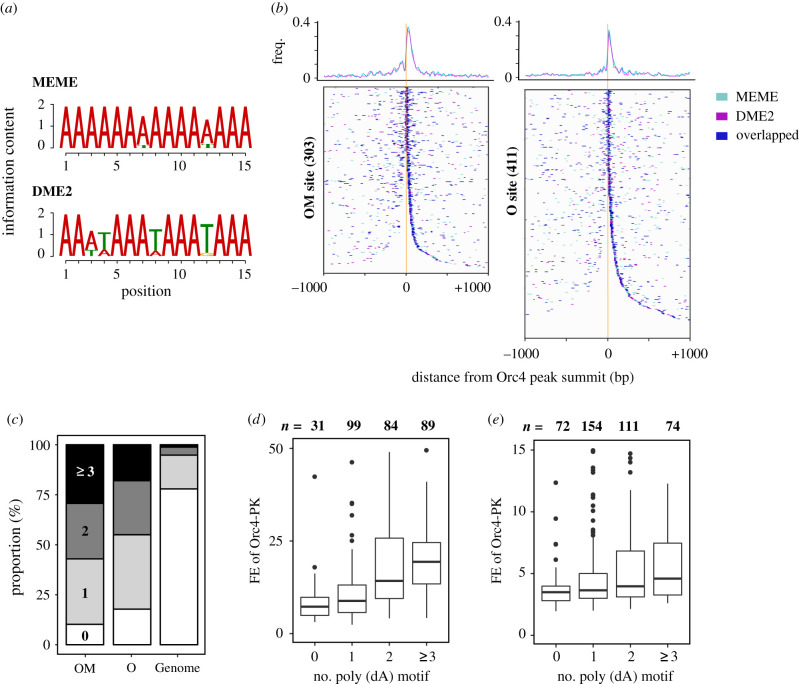
Poly(dA) motif is associated with Orc4-binding sites. (*a*) Sequence logos of DNA motifs that appeared frequently around Orc4-binding sites. Results obtained by two motif finders, MEME (top) and DME2 (bottom), are shown. (*b*) Position of the motifs around each OM (left) or O (right) site, relative to the Orc4 ChIP-seq peak summit. OM and O sites were sorted by distance to the nearest motif and oriented so that the nearest one was on the right side. Magenta, motif discovered by MEME; cyan, motif by DME2. Blue indicates a sequence that fits both motif signatures. Distribution profiles of the motifs were shown on the top. (*c*) Number of poly(dA) motifs (union of the motifs by MEME and DME2) around each OM and O site (±250 bp). Proportion of the sites possessing no, one, two and three or more sites is shown in white, light grey, dark grey and black, respectively. Genome, genome-wide average. (*d,e*) Correlation between Orc4 ChIP-seq FE at the OM (*d*) or O (*e*) sites and the number of motifs located around (±250 bp) the sites. The distribution of FE values for sites with the indicated motif number is shown as box plot.

We first searched for DNA motifs associated with the OM sites. MEME, a motif discovery tool [37], identified a 15 bp-long motif with a stretch of As (figure 2*a*) in the vicinity of the OM sites. Another tool, DME2 [38], also revealed a similar AT-rich, highly asymmetric motif (figure 2*a*). We then mapped the position of the two discovered motifs relative to Orc4 ChIP-seq peak summits, and found that they exhibited indistinguishable distributions and were located within a 100 bp window around the Orc4 peak summits at most of the OM sites (figure 2*b*). In addition, these two motifs coincided at many places. Therefore, we consider the two motifs discovered by MEME and DME2 to be slightly different representations of the same DNA signature and hereinafter refer to them collectively as the ‘poly(dA) motif’. The poly(dA) motif resembles the poly(dA) tracts that are found in several origins and crucial for replication activity [16–18], though shorter in length. The sequences of the poly(dA) motifs seen at each of the OM sites are summarized in electronic supplementary material, table S4.

One or more poly(dA) motifs were present within 250 bp of the Orc4 peak summits for greater than 90% of the OM sites, much more frequent than expected under the assumption of a random distribution (95% confidential interval [CI_95%_], 18–26%) (figure 2*c*). Notably, the poly(dA) motif exhibited a similar enrichment around the O sites (figure 2*b,c*). We also found that the number of motifs around the OM and O sites was positively correlated with FE of Orc4 in ChIP-seq (figure 2*d,e*). Taken together, these results strongly suggest that the poly(dA) motif ChIP-seq analysis revealed is indeed involved in Orc binding to DNA.

### 3.3. High AT content observed at Mcm-binding sites

**Figure 3.**
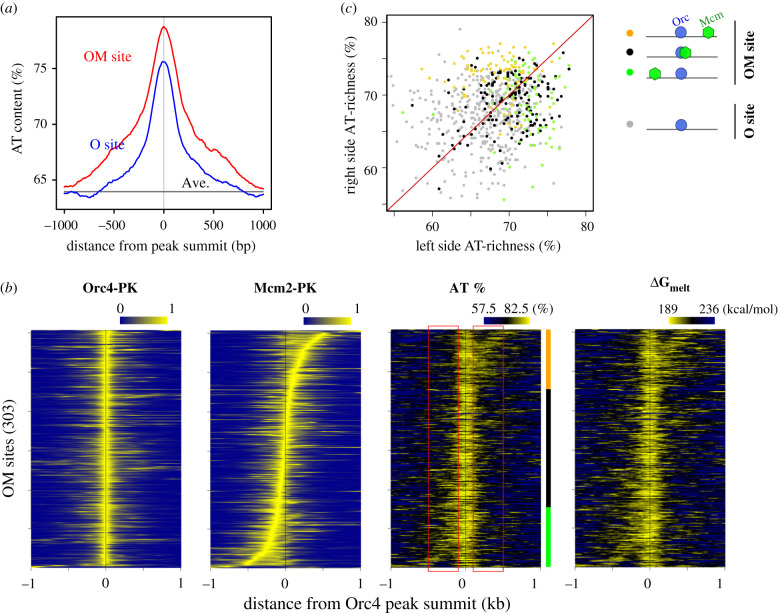
Mcm-binding sites are associated with an AT-rich DNA segment. (*a*) Averaged AT content (in %, 100 bp sliding window) profiles around OM and O sites. Ave., genome-wide average. (*b*) Heatmap representations of Orc4 and Mcm2 ChIP-seq profiles, as well as AT content (AT%) and *Δ*G_melt_ (the calculated energy required for local DNA melting) at each OM site, relative to the summit of Orc4 peak. The OM sites were sorted based on the distance between Orc4 and Mcm2 ChIP-seq peak summits. ChIP-seq profiles were scaled so that the local maximum became equal to 1. (*c*) Plot of AT-content values in the regions adjacent to Orc4 peak summit (left, −500 to −100 bp; right, +100 to +500 bp; indicated as red rectangle boxes in (*b*)) at each OM or O site. Orange, OM sites where the Mcm2 peak was shifted rightward relative to the Orc4 peak; black, OM sites where Orc4 and Mcm2 peaks overlapped; green, OM sites where the Mcm2 peak was shifted leftward (as indicated by a coloured vertical line in (*b*)). Grey, O sites.

We then assessed the base content of OM sites. Consistent with previous findings [25,26], we observed an increase in AT content at OM sites relative to the genomic average (figure 3*a*). The DNA sequences around the O sites were also AT-rich. However, the increase in AT content was greater in magnitude and affected a longer stretch of sequence around the OM sites, indicating that AT richness may be linked to the difference between the OM and O sites. To explore this point further, we compared the AT content profile with Orc- and Mcm-binding locations around each OM site (figure 3*b*). First, we found that the summits of Orc4 and Mcm2 ChIP-seq peaks were virtually overlapping for about half of these sites, while they were separated by 100–500 bp for the others. We then noticed that AT content increased around all Orc4 summits, presumably due to the presence of the poly(dA) motif. In addition, another approximately 200 bp DNA segment with relatively high AT content was evident at the peak summits of Mcm2 when they separate from those of Orc4. In figure 3*c*, we plotted AT contents of 400 bp DNA regions that were left- or right-adjacent to an Orc4 peak (−500 to −100 bp and +100 to +500 bp, respectively). The split OM sites exhibited significantly higher AT content on the side where Mcm was localized: if Mcm was located separately to the right side of Orc (orange dots), the AT content of the right-hand DNA segment was higher than that of the left-hand segment, and vice versa (green dots). The other OM site (black dots), where Orc and Mcm peak summits were very close, exhibited less difference in the AT content of the right and left segments; however, the AT contents of these regions were higher than those of the O sites (grey dots) to which Mcm was bound inefficiently. Therefore, we conclude that a few hundred base pair DNA segment with high AT content facilitates stable binding of Mcm to DNA.

AT-rich DNA is thought to denature easily. We estimated the energy required for local DNA melting (ΔG_melt_) around each OM site by the nearest-neighbour method [39] and confirmed that a decrease in ΔG_melt_ coincided with Mcm2 as well as Orc4 peak summits (figure 3*b*). This suggests that high AT content associated with Mcm-binding sites may promote local DNA unwinding.

### 3.4. Preferential localization of OM sites in long IGRs

**Figure 4.**
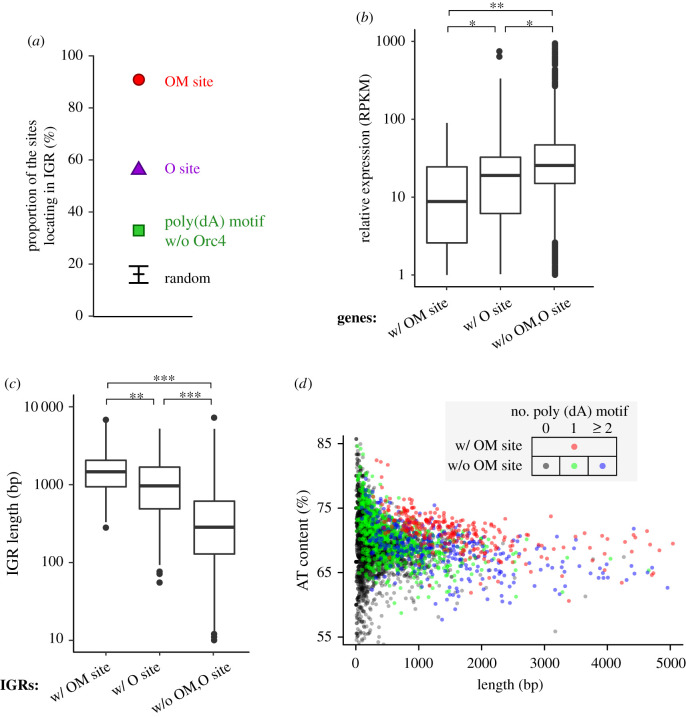
OM sites are preferentially located in long intergenic regions. (*a*) Proportion of sites located within intergenic regions (IGRs). Magenta circle, OM sites; purple triangle, O sites; green square, poly(dA) motif sites not associated with OM or O sites. Black line and error bars indicate mean and CI_95%_ for randomly sampled genomic sites, respectively. (*b*) G1-phase expression levels of genes containing OM sites, O sites, or neither OM nor O sites. The numbers of the corresponding genes are 28, 173 and 5,144, respectively. *, *p* < 10^−3^; **, *p* < 10^−6^ (Mann–Whitney *U*-test). (*c*) Lengths of IGRs containing OM sites, O sites, or neither OM nor O sites. The numbers of the corresponding IGRs are 268, 175 and 3,284, respectively. **, *p* < 10^−6^; ***, *p* < 10^−15^ (Mann–Whitney *U*-test). (*d*) Scatter plot representation of length and AT content of each IGR. IGRs containing OM sites are indicated in red. OM site-negative IGRs containing no, one and two or more poly(dA) motifs are shown in black, green and blue, respectively.

The *S. pombe* genome is relatively compact and mostly contains genes separated by short intergenic regions (IGRs) with a median size around 300 bp. However, previous studies [25,26,53,54] showed that fission yeast replication origins were preferentially located in IGRs. Consistent with this, we found that 91% of OM sites and 56% of O sites in our dataset were located in IGRs (figure 4*a*), many more than would be expected by chance (CI_95%_, 13–20%). For the remaining 9% OM sites, which were located within genes, we recognized that the corresponding genes were less transcriptionally active than the genome-wide average (figure 4*b*). Hence, we conclude that OM sites tend to be located in transcriptionally inactive regions. We also observed that the IGRs with OM sites were significantly longer than those containing neither OM nor O sites (figure 4*c*; median lengths of 1466 and 277 bp, respectively). Together, these data suggest that the length of a transcriptionally inactive region is also an important factor in facilitating pre-RC assembly on chromosomes.

### 3.5. Preferred gene orientation of OM site-containing IGRs

**Figure 5.**
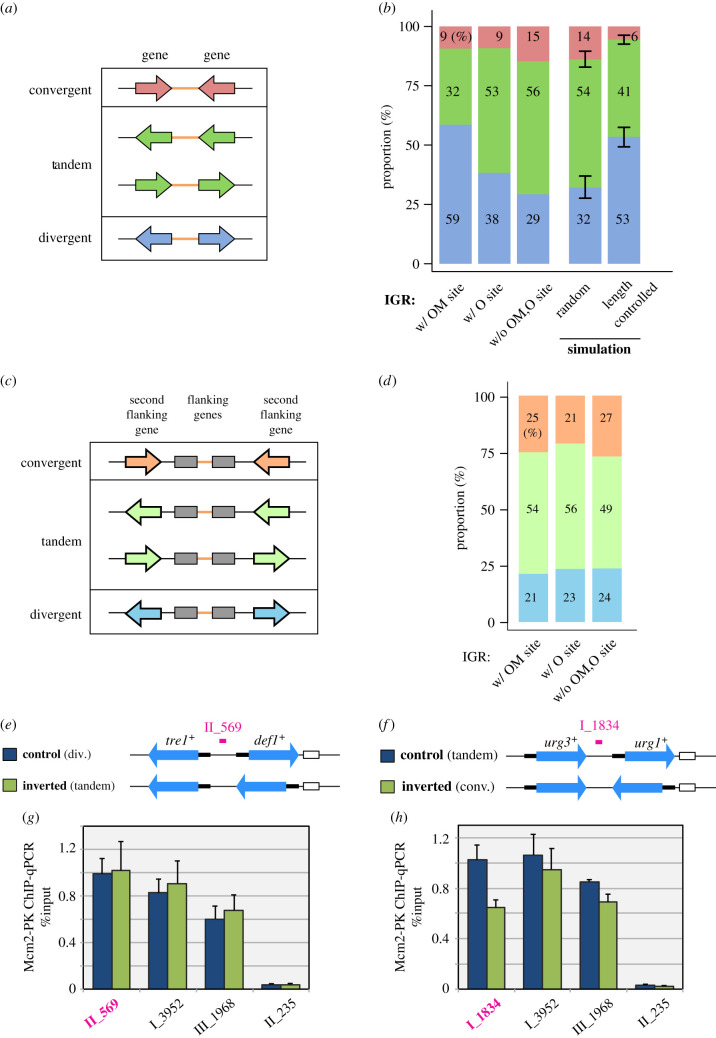
Correlation between OM site presence and gene orientation. (*a*) Classification of IGRs into convergent, tandem and divergent types, based on orientation of the flanking genes. (*b*) Proportion of each gene orientation type for IGRs with OM sites, with O sites and without either OM or O sites. Red, convergent; green, tandem; blue, divergent. Results of Monte Carlo simulation are also shown. In ‘random’, IGRs were randomly picked from the genome, whereas in ‘length controlled’, IGRs were picked so that they had the same length distribution as that observed for IGRs with OM sites. Error bars, CI_95%_. (*c*) Another IGR classification, based on orientation of the second flanking genes. (*d*) Proportion of the second flanking gene orientation types for IGRs with OM sites, with O sites and with neither OM nor O sites. Pale red, convergent; pale green, tandem; pale blue, divergent. (*e–h*) Gene inversion experiments. (*e*,*f*) Strains used for experiments. By inverting *def1*^+^ (*e*) and *urg1*^+^ (*f*), gene orientation type of the adjacent IGR was changed (divergent to tandem in (*e*), tandem to convergent in (*f*)). Black thick line, promoter. White rectangle, a marker gene used for strain construction. (*g*,*h*) qPCR measurement of Mcm2-PK bound to the indicated genomic loci in the gene-inverted and control strains arrested in G1 phase. Loci in magenta correspond to the IGRs with gene inversion. I_3952 and III_1968 are IGRs with OM sites. II_235 is an IGR without Mcm2 binding. Means with error bars (SD) from three biological replicates are shown.

On the basis of the transcription orientation of the flanking genes, IGRs can be classified into three types: divergent, tandem and convergent (figure 5*a*). Segurado *et al*. [25] observed that relatively long IGR with high AT content, in which origins are frequently mapped, are overrepresented in divergent IGRs. We confirmed that OM site-positive IGRs showed the same preference for divergent orientation; 59% of OM site-positive IGRs were divergent type, much more frequent than expected under the assumption of a random distribution (CI_95%_, 28–37%) (figure 5*b*). Because highly transcribed genes are aligned codirectionally with replication in prokaryotes [55], we wondered whether this could also be the case for the fission yeast genome. However, this turned out not to be correct, because the second-nearest genes to OM sites exhibited no bias in orientation (figure 5*c*,*d*), and we observed no difference in expression levels between divergent genes and genes with other orientations located adjacent to OM sites (data not shown). We then noticed that the biased orientation could mostly be attributed to the afore-mentioned preferred localization of OM sites in long IGRs. In *S. pombe*, IGRs with a divergent orientation were longer than those in tandem or convergent orientations. When IGRs were chosen randomly but in such a way that their length distribution coincided with that of OM site-positive IGRs, they had a gene orientation profile very close to that observed for the OM site-positive IGRs, with a preference for divergent orientation (expected proportion of divergent IGR is 49–58% [CI_95%_]) (figure 5*b*).

We then experimentally tested whether gene orientation plays a role in facilitating/inhibiting pre-RC assembly. For this purpose, we chose the IGRs between *tre1*^+^ and *def1*^+^ genes and between *urg3*^+^ and *urg1*^+^ genes as representative IGRs with an OM site. They are divergent and tandem types, respectively. By inverting the orientation of *def1*^+^ and *urg1*^+^, we changed the former IGR to tandem orientation, and the later to convergent one, and investigated how the gene orientation change affected assembly of pre-RC at these sites by ChIP-qPCR of Mcm2 (figure 5*e–h*). We found that the divergent-to-tandem change did not affect chromosomal binding of Mcm2 in *cdc10*-arrested cells (figure 5*e*,*g*), indicating that divergent orientation is dispensable for pre-RC assembly. The tandem-to-convergent change also did not abolish Mcm2 binding, but we observed that the amount of Mcm2 bound to the corresponding IGR was consistently reduced by approximately 40% (*p* < 0.05) (figure 5*f*,*h*). These results imply that gene orientation is not a primary requirement for pre-RC assembly, but may be an auxiliary factor and affect efficiency of Mcm loading process.

### 3.6. Three DNA-encoded features that are sufficient to specify OM site location

Our analysis so far revealed association of OM sites (or efficient replication origins) with three characteristics in the genome: the poly(dA) motif, a highly AT-rich DNA segment, and a transcriptionally inactive DNA region. We also considered other features, such as the expression level of the surrounding genes and the distance to various genomic landmarks, but these exhibited little, if any, correlation with the presence of OM sites. In a plot of AT content versus length for all IGRs (figure 4*d*), OM site**-**containing IGRs were distributed separately from IGRs without OM sites. This indicates the importance of these characteristics in specification of replication origin, as previously reported [25,26].

**Figure 6.**
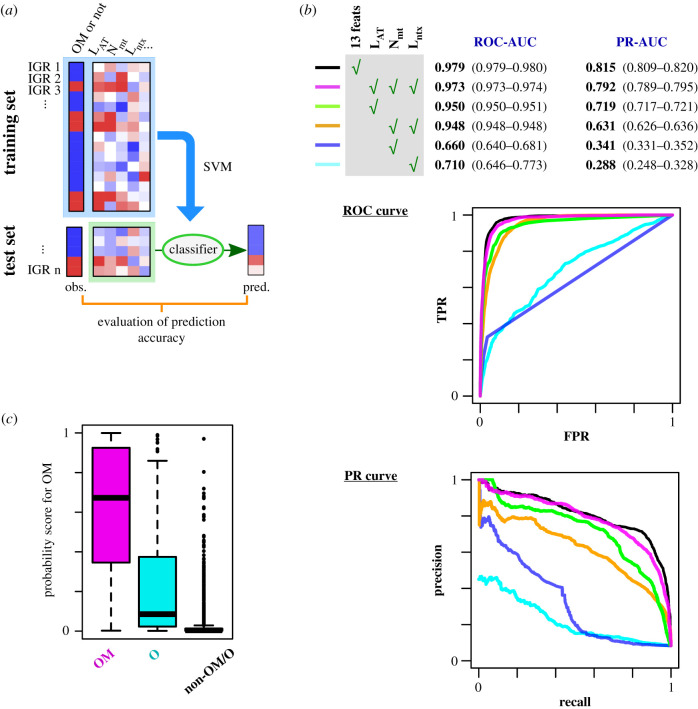
DNA-encoded features are sufficient to specify OM site location. (*a*) An outline of classifier building and evaluation. (*b*) ROC and PR curves of classifiers based on the indicated features. The averaged result of ten times repeated fourfold cross validation is shown. Numbers shown at the top are the means and 95% confidence intervals (in parentheses) of AUC. 13 feats, all the 13 features listed in electronic supplementary material, figure S2a. TPR, true-positive rate; FPR, false-positive rate. AUC, area under the curve. (*c*) Box plot of the probability scores calculated for IGRs that actually contain OM sites (OM), O sites (O), and neither OM nor O sites (non-OM/O). The used classifiers were trained on *L*_AT_, *N*_mt_ and *L*_ntx_.

If the abovementioned three characteristics play a major role in specifying replication origins, the origin sequences can be distinguished from the rest of the genome computationally by using these features. To test this idea rigorously, we trained probabilistic classifiers based on support vector machine (SVM) machine learning algorithm [42,45], using multiple features at 1 kb DNA regions centred on each IGR as predictors. The features computed for the analysis are related in some way to one of the three characteristics (the poly(dA) motif, AT richness and a transcription-free region) and summarized in electronic supplementary material, figure S2a. We also included two additional features that are related to neighbouring gene orientation. The DNA fragments in the dataset were randomly divided into training and test sets, and the training set was used to train an SVM classifier that takes the features as parameters and returns the probability of being an OM site-containing IGR (figure 6*a*). The performance of the trained classifier was evaluated using the test set that was not seen by the classifier (figure 6*a*; see details in Methods).

The classifiers predicted high probabilities of OM site presence specifically for the IGRs that actually contained OM sites (electronic supplementary material, figure S2b). The results were presented as a receiver operator characteristic (ROC) curve and precision-recall (PR) curve, in which pairs of the true-positive rate (TPR) and the false-positive rate (FPR) and pairs of precision and recall values, respectively, were computed and plotted while changing the threshold value (figure 6*b*, black lines). The ROC curve is widely used in evaluation of classifier performance, while the PR curve is more adequate to evaluate models for imbalanced dataset like this case (i.e. the current dataset consists largely of OM site-negative IGRs). The plots demonstrate that both high TPR and low FPR, and both high precision and high recall values were simultaneously attained, indicating accurate discrimination by the classifiers. The area under the curve of ROC and PR curves (ROC-AUC and PR-AUC), which are performance metrics and equal 1 for perfect classifiers, were 0.979 and 0.815, respectively. These results revealed that the presence of an OM site in an IGR can be predicted by the used 13 features.

We then attempted to identify the features that are essential for accurate prediction. Each feature was evaluated by calculating F-score, which describes how effective the feature is in discrimination of OM site-positive IGRs from the others (electronic supplementary material, figure S2c) [48]. With help of this information, we found that the following three features were sufficient to generate classifiers that perform almost as accurately as ones based on all 13 features: (i) the number of poly(dA) motifs (*N*_mt_), (ii) the length of AT-rich regions where local (101 bp window) AT content was 0.75 or higher (*L*_AT_) and (iii) the length of the transcription-poor region where the mapped RNA-seq read count is ≤1 (*L*_ntx_). The ROC-AUC and PR-AUC of the classifiers based on *N*_mt_, *L*_AT_, and *L*_ntx_ reached 0.973 and 0.792, respectively (figure 6*b*, magenta). The features linked to neighbouring gene orientation were excluded without affecting prediction accuracy, supporting the abovementioned conclusion that gene orientation plays only a minor role in origin location specification. We also noticed that the classifiers assigned low-to-moderate level of probability scores to O site**-**containing IGRs (figure 6*c*).

The same classifiers trained on the three features of IGR DNA sequences were used to distinguish intragenic OM sites from control DNA fragments selected randomly from across the entire genome. We found that classification performance was very high (ROC-AUC 0.989, PR-AUC 0.948). This demonstrates that efficient pre-RC assembly sites in intragenic and intergenic regions share very similar attributes of DNA sequence.

### 3.7. Each of *N*_mt_, *L*_AT_ and *L*_ntx_ is required for specifying OM sites

As mentioned above, AT-richness is known to be a good indicator of the replication origin's position in fission yeast genome. Consistently, we found that the classifier trained only on *L*_AT_ performed with substantial accuracy (ROC-AUC 0.950, PR-AUC 0.719) (figure 6*b*, green). By contrast, classifiers trained only on *N*_mt_ or on *L*_ntx_ exhibited poor performance (figure 6*b*, blue and cyan lines, respectively). However, PR curve analysis indicated that the classifiers trained on *L*_AT_ alone were significantly less accurate than those trained on *N*_mt_, *L*_AT_, and *L*_ntx_, indicating that *L*_AT_ is not a single essential feature for OM site prediction.

**Figure 7.**
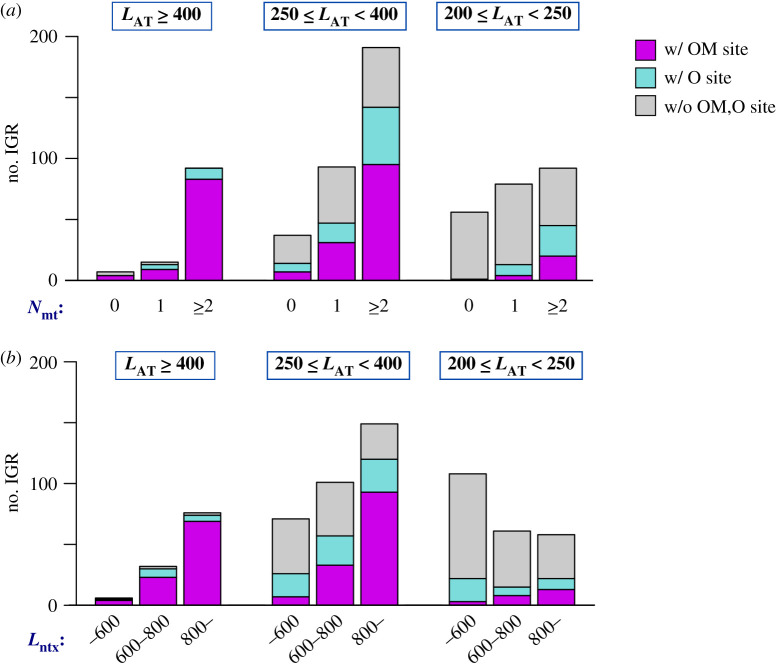
Each of *L*_AT_, *N*_mt_ and *L*_ntx_ shows correlation with OM site presence. (*a,b*) Stacked histograms of IGRs with the indicated *L*_AT_ and *N*_mt_ values (*a*), and *L*_AT_ and *L*_ntx_ values (*b*). Magenta, IGR with OM sites; cyan, IGR with O sites; grey, IGRs without OM or O sites.

We, then, asked why *L*_AT_ behaved as the most important feature and found that both *N*_mt_ and *L*_ntx_ were partially dependent on *L*_AT_. When we extracted IGRs with high *L*_AT_ value (*L*_AT_ ≥ 400), most of them possessed one or more poly(dA) motif (*N*_mt_ ≥ 1) and showed high *L*_ntx_ value (*L*_ntx_ ≥ 600) (figure 7*a,b*). Hence, the IGRs with high *L*_AT_ value concurrently possessed sufficiently high values of *N*_mt_, and *L*_ntx_ and fulfilled the requirements of becoming the OM sites. Importance of *N*_mt_ and *L*_ntx_, however, becomes apparent when focusing on IGRs with lower *L*_AT_ value. IGRs with an intermediate level of *L*_AT_ value (250 ≤ *L*_AT_ < 400) showed wider ranges of *N*_mt_ and *L*_ntx_ values, and both of them exhibited significant correlation with the probability of OM site presence (figure 7*a,b*). With even lower *L*_AT_ value (200 ≤ *L*_AT_ < 250), only few IGRs contained OM sites. Consistent with the notion that *L*_AT_ alone is not an essential feature for OM-site classification, SVM classifies trained on *N*_mt_ and *L*_ntx_, but not *L*_AT_, revealed considerably good classification performance (ROC-AUC 0.948, PR-AUC 0.631) (figure 6*b*, orange). Taken together, the results strongly suggest that all three features, *N*_mt_, *L*_AT_ and *L*_ntx_, play a role in specification of pre-RC assembly sites in the fission yeast genome.

## 4. Discussion

In this work, we investigated features of *S. pombe* replication origins, based on ChIP-seq data of the pre-RC components Orc4 and Mcm2. About 300 pre-RC binding sites, or OM sites, were detected in the fission yeast chromosome arms in *cdc10*-arrested cells, in which Cdc18 and Cdt1 were not induced. Efficient replication origins identified in a previous work [51] were found to be highly enriched in the OM sites. Presumably, basal expression of Cdc18 and Cdt1 is sufficient to promote pre-RC assembly at efficient origins, at least when G1 phase is prolonged. Intriguingly, high-resolution ChIP-seq profiles revealed that Mcm-binding location was apart from that of Orc by a few hundred bp at approximately 50% of the OM sites. The profiles also uncovered a significant number of Orc-only sites (or O sites), to which Mcm loading is inefficient, on the chromosomes. We used these findings and unveiled the attributes that were associated closely with Orc- or Mcm-binding sites.

We found that the poly(dA) motif, which consists of clusters of homopolymeric A residues, frequently appeared at OM and O sites. The poly(dA) motif resembles the sequence to which *S. pombe* Orc4 exhibits high affinity *in vitro* and *in vivo* [19–23]. This binding depends on the AT hooks, which Orc4 in *S. pombe* and a related yeast uniquely possesses [19,20]. Consistent with the notion that the poly(dA) motif is the binding sequence of Orc4, the motif was located very near the Orc4 ChIP-seq peak summits and was found at both OM and O sites, regardless of Mcm helicase binding (figure 2*b*). Similar DNA signatures were previously reported to be enriched in the vicinity of replication origins [24]. Our result remonstrated that the poly(dA) motif is a general feature of Orc-binding sites in fission yeast replication origins.

Our analysis revealed an approximately 200 bp AT-rich segment as another feature associated with the origins. This feature was colocalized with the Mcm-binding site within the origins and played a crucial role in discrimination of OM sites from O sites (figure 3). Consistently, the features related with AT richness, but not with the poly(dA) motif, showed higher F-scores in discrimination between OM site-positive IGRs and the others, than between O site-positive IGRs and IGRs without OM or O sites (electronic supplementary material, figure S2c). The AT-rich segment resembles Region II of *ori2004*, whose deletion impedes efficient binding of Mcm, but not Orc, to this origin [23]. Our result has extended their finding and suggests that it is generally required for efficient Mcm binding in fission yeast origins. Previous genome-wide studies [25,26] demonstrated that fission yeast replication origins can be identified as approximately 1 kb-long IGRs with significantly high AT content. Our analysis strongly suggests that the AT-rich nature of fission yeast replication origins is actually attributed to two distinct features, the poly(dA) motif and AT-rich region of a few hundred bp, and both of them contribute independently to origin specification in the genome. Highly AT-rich IGRs (IGRs with high *L*_AT_) in the genome almost always contain one or more poly(dA) motif and possess a long transcription-poor region (figure 7). This explains why *L*_AT_ on its own acts as a reasonably good predictor of replication origin. However, analysis of IGRs with moderate *L*_AT_, in which the other two features *N*_mt_ and *L*_ntx_ are more independent variables, confirmed that *N*_mt_ and *L*_ntx_ also play an important role in OM site prediction (figure 7).

As mentioned above, several studies reported that fission yeast origins are frequently located in approximately 1 kb-long IGRs [25,26,53,54], which we confirmed in figure 4. In addition, we found that, if an OM site was located in a gene, the corresponding gene tended to be transcriptionally inactive. Transcription across an origin may be detrimental to pre-RC assembly. In budding yeast, the origin activity of *ARS605*, which is located within the *MSH4* gene, is abolished specifically when *MSH4* gene is expressed in meiotic S-phase [56]. Detrimental effect of transcription on pre-RC assembly may be universal across species. The dimensions of reconstituted budding yeast pre-RC [57] suggest that the length of DNA covered with pre-RC is 100 bp or less. It remains to be elucidated why much longer DNA region with poor transcription is necessary for efficient pre-RC assembly in fission yeast.

Machine learning-based modelling demonstrated that a combination of the three features (the poly(dA) motif, an AT-rich region of a few hundred bp, and a transcriptionally inactive region of approximately 1 kb), enabled accurate discrimination of the OM sites from the rest of the genome. These three features are, hence, distinct characteristics of efficient origins in fission yeast. The classifiers successfully gave low-to-moderate probability scores to the O sites, or inefficient pre-RC assembly sites, suggesting that the three features are important parameters to determine origin activity of a given DNA sequence. We propose that the three features are the major *cis*-determinants of fission yeast replication origins, which awaits experimental validation. A limitation of this study is that we investigated only natural replication origins in the genome. Cotobal *et al*. [58] reported that an artificial highly AT-rich DNA as short as 100 bp can be an origin in a chromosomal context. Extended analysis of diverse variety of synthetic sequences would help clarify further the requirements for being an origin.

The current study revealed that fission yeast replication origins resemble those of budding yeast and *E. coli* in that an origin is comprised multiple elements, including a recognition site of origin binding protein and additional *cis*-elements. Guided by this analogy, we propose three models of how the AT-rich segment functions in pre-RC assembly at fission yeast replication origins. (i) As in budding yeast, the AT-rich segment may result in nucleosome depletion, thereby contributing to pre-RC formation. *In vivo* chemical mapping of nucleosome, however, revealed that AT-rich sequence in *S. pombe* genome does not disfavour nucleosome occupancy, and that nucleosome density is only slightly lowered at replication origins [59]. (ii) The AT-rich segment may serve as a DNA unwinding element. A similar element is known to be required for replicative helicase recruitment to origins in *E. coli* [60]. A structural study of budding yeast Mcm [61] suggested that DNA within Mcm double-hexamer, or Mcm complex in the pre-RC, is indeed kinked and partly melted. (iii) The AT-rich segment may function as a secondary Orc-binding site. Recent biochemical study of budding yeast origins suggested that additional Orc-binding sites on the opposite strand of the ACS is required for efficient loading of Mcm helicases [62]. The AT-rich segment in fission yeast origins may serve as degenerate poly(dA) motifs and promote transient Orc binding. These three models are not necessarily mutually exclusive. Further investigation is required to clarify the role of AT-rich segment in *S. pombe* origin specification.

## Supplementary Material

Supplementary Tables

## Supplementary Material

Supplementary Figure S1

## Supplementary Material

Supplementary Figure S2

## Data accessibility

ChIP-seq and whole-genome sequencing data from this study are available from the Gene Expression Omnibus database under the accession number GSE125335.
