# Supplementary material for: Bioinformatical dissection of fission yeast DNA replication origins
Source: Open Biol. 2020 Jul 22;10(7):200052. doi: 10.1098/rsob.200052 (PMC7574548; doi:10.1098/rsob.200052)
Supplement: Supplementary Tables [file rsob200052supp1.pdf]

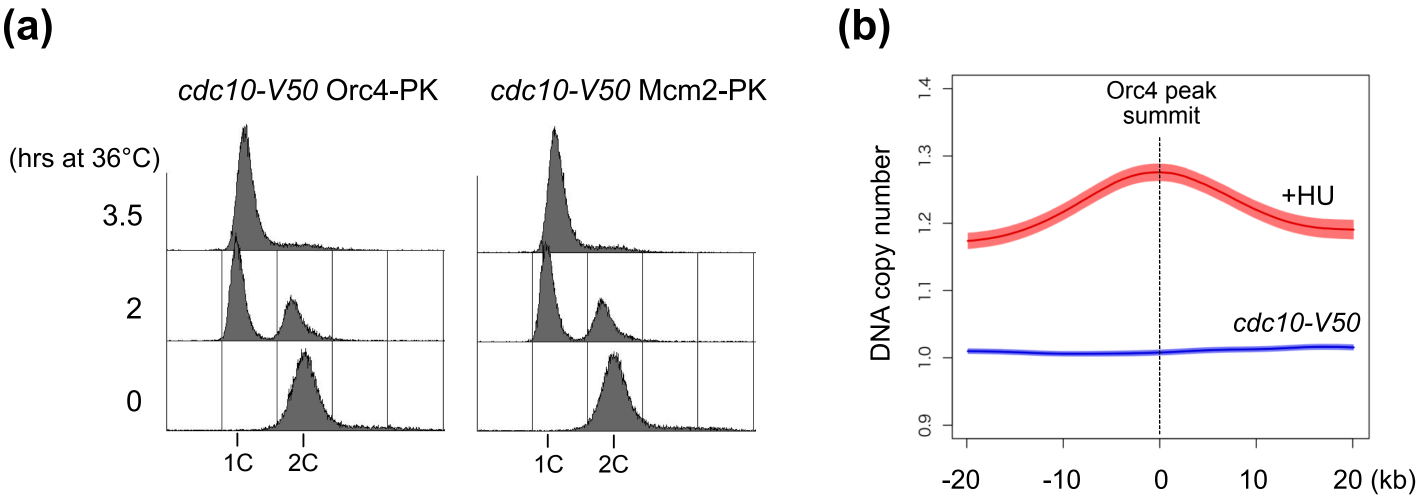

(a) Flow cytometry of the cells used for ChIPseq. (b) Aggregation plot of DNA copy number profile centered on Orc4 ChIP-seq peak summits. Red, cells arrested in early S ohase by HU. Blue, cells arrested by *cdc10-V50* mutation. Lines in dark color indicate the mean, while areas in light color the SEM.

Supplementary Figure 1
