# Supplementary material for: Bioinformatical dissection of fission yeast DNA replication origins
Source: Open Biol. 2020 Jul 22;10(7):200052. doi: 10.1098/rsob.200052 (PMC7574548; doi:10.1098/rsob.200052)
Supplement: Supplementary Figure S1 [file rsob200052supp2.pdf]

(a)

Poly(dA) motif

1. Number of motif **(= N_{mt})**

2. Presence of motif (presence = 1, absence = 0)

3. AT-skewness for the entire sequence

4. Maximum of local AT-skewness

5. Total length of the regions where local AT-skewness is >0.5 (bp)

AT-richness

6. AT-richness for the entire sequence

7. Maximum of local AT-richness

8. Length of the regions where local AT-richness is >0.75 (bp) **(= L_{AT})**

9. Length of the regions where local AT-richness is >0.8 (bp)

Transcription

10. Mean of mapped RNA-seq read count at each bp

11. Total length of the regions where mapped RNA-seq read count is 1 or less (bp) **(= L_{ntx})**

Neighboring gene orientation

12. Indicator variable for divergent orientation (div. = 1, else = 0)

13. Indicator variable for tandem orientation (tandem. = 1, else = 0)

(b)

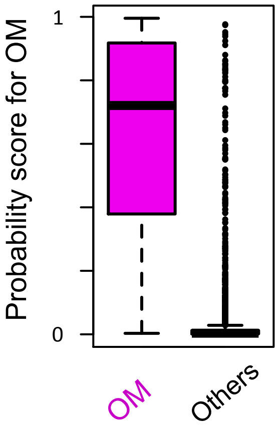

(c)

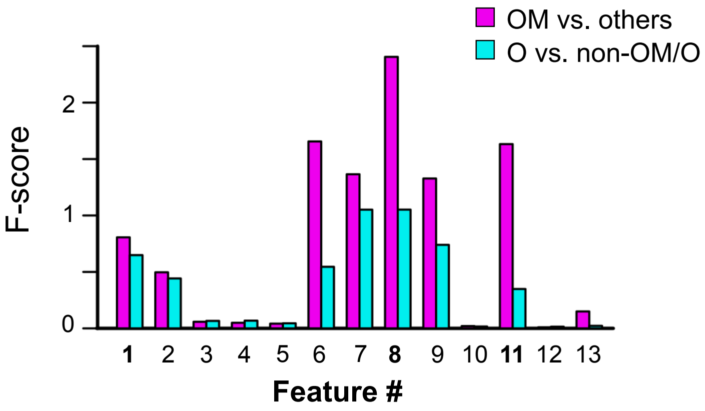

(a) Features used for SVM-based classifier building. All features were calculated for 1 kb DNA fragments corresponding to each IGR. Local AT-richness and skewness were calculated using a 101-bp long sliding window. (b) Box plot of the probability scores calculated for IGRs that actually contain OM sites (OM) and the others. The used classifier was trained on all the 13 features. (c) F-score (Chen and Lin, 2006) for each of the 13 features. Magenta, discrimination of OM–positive IGRs and the other IGRs. Cyan, discrimination of O–positive IGRs and IGRs without OM or O sites.

Supplementary Figure 2
